# Extracellular Vesicles and Their Impact on the Biology of Protozoan Parasites

**DOI:** 10.3390/tropicalmed8090448

**Published:** 2023-09-15

**Authors:** Manu Sharma, Daniela Lozano-Amado, Debabrata Chowdhury, Upinder Singh

**Affiliations:** 1 Division of Infectious Diseases, School of Medicine, Stanford University, Stanford, CA 94305, USA; msharma4@stanford.edu (M.S.); dlamado@stanford.edu (D.L.-A.); debc2021@stanford.edu (D.C.); 2 Department of Microbiology and Immunology, School of Medicine, Stanford University, Stanford, CA 94305, USA

**Keywords:** protozoa, extracellular vesicles, immune response, intercellular communication

## Abstract

Extracellular vesicles (EVs) are lipid-membrane-bound structures produced naturally by all cells and have a variety of functions. EVs act as vehicles for transporting important molecular signals from one cell to another. Several parasites have been shown to secrete EVs, and their biological functions have been extensively studied. EVs have been shown to facilitate communication with the host cells (such as modulation of the host’s immune system or promoting attachment and invasion into the host cells) or for communication between parasitic cells (e.g., transferring drug-resistance genes or factors modulating stage conversion). It is clear that EVs play an important role in host–parasite interactions. In this review, we summarized the latest research on the EVs secreted by protozoan parasites and their role in host–parasite and parasite–parasite communications.

## 1. Introduction

Protozoan pathogens are responsible for many diseases in both humans and animals, accounting for a significant economic burden globally. These unicellular parasites surmount several challenges in establishing a successful infection. In their effort to survive in the host environment, one approach that the parasites utilize is the secretion of extracellular vesicles (EVs) to communicate with each other or to interact with distal host cells. The transport of biologically active molecules through EVs can modulate the behavior of the recipient cells, which can be beneficial for the parasite in adhering to and infecting the host [1]. However, at other times, the host’s immune cells internalize the parasitic EVs, and this primes the immune cells against the infection [2].

EVs are lipid-bilayer-bound vesicles that contain a selectively packaged cargo of proteins, DNA, RNA, and lipids [3,4]. They are heterogenous and vary in size, morphology, and composition depending on the type of cell and the environment. To maintain conformity, the International Society for Extracellular Vesicles (ISEV) defines EV as a collective term for “particles naturally released from the cell that is delimited by a lipid bilayer and cannot replicate” [5,6,7]. 

Particle tracking analysis has shown heterogeneity in the size of the vesicles released by cells. EVs have been classified according to their biogenesis and size. Exosomes are typically 50–150 nm in size, originate from the endosomal membrane, and are released into the extracellular environment when multivesicular bodies fuse with the plasma membrane [4]. Microvesicles are bigger than exosomes, with diameters ranging from 200 nm to 1 µm; they are released by budding from the plasma membrane [8]. Protozoan parasites have been reported to shed both exosome-like vesicles and larger microvesicle-like vesicles. For example, *Trichomonas vaginalis* has been reported to release vesicles with a size ranging from 40 nm to 1000 nm [9].

However, as well as the variance in their size, there is also heterogeneity in the EVs’ cargo. As the contents of EVs have been characterized in depth with newer lipidomic, proteomic, and RNA sequencing techniques, it has become clear that EVs’ cargoes differ depending on the parental cell type and the culture conditions [10]. Protozoan parasites have also been reported to control the cargo being packaged in their EVs to modulate the specific signal sent to the recipient cells. 

Additionally, intracellular stages of parasites, such as *Leishmania* or *Plasmodium*, have the ability to modulate the cargo of the EVs secreted by the infected host cells to promote proliferation and pathogenesis. Interestingly, viruses have been found inside the EVs secreted from some cells [11]. This has been seen in parasites as well. Atayde et al. discovered that *Leishmania* EVs are used as a viral envelope by the *Leishmania* RNA Virus 1, facilitating transmission. Similarly, Rada et al. reported that *Trichomonas* virus particles are released from *T. vaginalis* cells within EVs [12,13]. 

The selective packaging of effector molecules into lipid-membrane-bound vesicles, which protect the cargo from external insults, makes EVs the ideal vehicle for intercellular communication [14]. Recent research has identified small RNA such as tRNA fragments and tRNA halves, which are enriched in EVs and could have a potential role in regulating gene expression in the target cells [15,16]. 

**Figure 1:**
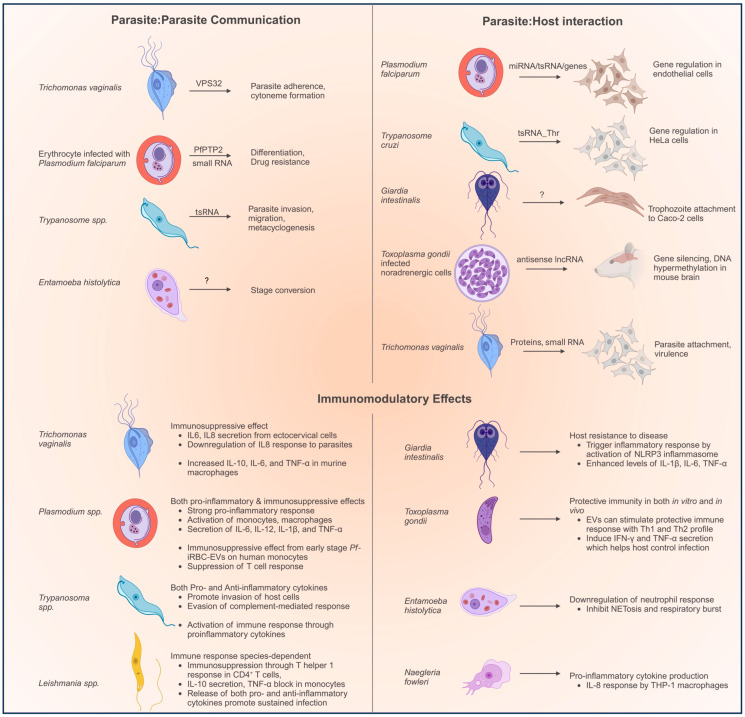
Examples of the biological functions mediated by EVs.

Parasites use EVs to transmit biologically active molecules to enable (a) parasite–parasite communication to help coordinate a concerted action, (b) parasite–host communication to facilitate adherence and virulence, and (c) parasite–host communication targeting the immune cells to suppress the immune response (Figure 1). At the same time, the host’s immune cells can intercept pathogenic EVs by recognizing pathogen-associated molecular patterns (PAMPs) through pattern recognition receptors (PRRs). PRRs include the family of Toll-like receptors (TLR) and nucleotide-binding oligomerization domain (NOD)-like receptors [17]. 

Thus, EVs play a central role in the interaction between the parasites and the immune system, which results in a spectrum of outcomes that encompass both pro- and anti-inflammatory effects. This complex interplay ultimately shapes the overall impact on the host’s health and the outcome of the parasitic infection. 

In the following sections, we discuss the features of the EVs secreted by different protozoan parasites in terms of their morphology, protein/RNA cargo, and their main biological functions. We summarize the functions of EVs in Figure 1. 

## 2. Extracellular Vesicles Secreted by Protozoan Parasites

### 2.1. Trichomonas vaginalis

*Trichomonas vaginalis* is responsible for the most common nonviral sexually transmitted infection worldwide, known as trichomoniasis. This disease leads to complications such as vaginitis, cervicitis, urethritis, and pelvic inflammatory disease, and increases the risk of transmitting HIV. Trichomoniasis has also been associated with the higher incidence and severity of cervical and prostate cancers [18,19,20].

*T. vaginalis* trophozoites colonize the human urogenital tract, where they adhere extracellularly to the epithelial cells. The EVs secreted by *T. vaginalis* are some of the best characterized parasitic EVs. Twu et al. first reported the secretion of exosome-like vesicles (Tv-ELVs) from the adherent B7RC2 strain of *T. vaginalis* [21]. Electron microscopy revealed that Tv-ELVs had a cup-shaped morphology with a size of around 50–150 nm. A different study reported that larger vesicles were also secreted by these parasites. Vesicles with diameters ranging from 100 to 1000 nm were termed “microvesicle-like structures” (MVs) and those larger than 1 µm were termed “large vesicles” (LVs). Electron microscopy showed that the MVs were being shed from the flagellar membrane of the parasite. Both MVs and LVs were found to be secreted in response to stimulation by the host cells and were selectively enriched for individual characterization of their proteomes. 

The Tv-ELVs’ proteome was characterized, and around 73% of the Tv-ELVs’ proteins were found to be orthologs of mammalian exosome proteins [21]. A member of the ESCRT III complex, VPS32, was shown to be important for biogenesis and the sorting of the protein cargo into the EVs (the protocol used did not separate the vesicles on the basis of their size and therefore, the generic term EV was used). Overexpression of VPS32 in the *T. vaginalis* trophozoites increased the secretion of EVs and improved the binding and uptake by the host cells [21].

Characterization of the RNA cargo showed small RNAs ranging in size from between 25 and 200 nt in the Tv-ELVs [21]. A later study showed that small RNAs made up ~55% of the total RNA, and tRNA fragments constituted the majority of these small RNA. Interestingly, the most abundant tRNA fragments were 5′ tRNA halves. *T. vaginalis* does not have miRNA genes, and tRNA halves could be important regulators of gene expression in these parasites [22]. Their presence in the Tv-ELVs suggests a possible role in parasite–parasite and parasite–host communications through gene regulation. However, the enrichment of tRNA halves due to nutrient deprivation such as serum starvation during the preparation of EVs cannot be ruled out. Further work is required to elucidate the role of the tRNA fragments packaged in the Tv-ELVs. 

Trichomonas parasites can harbor an double-stranded RNA virus endosymbiont, *Trichomonasvirus*, TVV. Govender et al. showed that TVV-positive parasites secreted EVs that carried a modified protein cargo that helped the parasite subvert the host’s immunity. [23]. However, recently, Rada et al. reported that the viral particles from TVV loaded inside *T. vaginalis* EVs resulted in a markedly higher proinflammatory immune response [13]. Future work should elucidate the role of the viral particles in promoting inflammation-related pathogenesis during trichomoniasis. 

Furthermore, the selective packaging of certain proteins involved in pathogenesis, such as some proteases, as well as surface proteins involved in parasite attachment was also observed. Rai et al. showed that 4-α-glucanotransferase (Tv4AGT) on the surface of Tv-ELVs binds to the heparan sulfate (HS) present on the host cell’s surface proteoglycans. This interaction was shown to be critical for the internalization of EVs through a lipid raft-mediated endocytosis process [24]. 

After adhering, Tv-ELVs fuse with the host cells to deliver their cargo into the cells. Once internalized by the recipient cells, these vesicles can modulate their behavior in various ways. Tv-ELVs can modulate both parasite–parasite and parasite–host cell adherence. Data have shown that pathogenic factors specific to parasitic strains were specifically packed into Tv-ELVs, and thus highly adherent strains of *T. vaginalis* secreted EVs that helped increase the attachment of a less adherent parasite strain to the host cells [21].

Internalization of the Tv-ELVs by ectocervical cells elicited the secretion of IL-6 and, to a lesser extent, IL-8 from the ectocervical cells. Preincubation of the cells with Tv-ELVs prior to the addition of *T. vaginalis* parasites led to a significant inhibition of the secretion of IL-8 but not IL-6 by the cells, indicating that Tv-ELVs specifically modulate the production of IL-8 by the ectocervical cells [21]. 

An immunomodulatory role of Tv-ELVs was found in an *in vivo* murine model of trichomoniasis [25]. *T. vaginalis* ELVs dampen the inflammatory response both *in vitro* and *in vivo*. Anti-inflammatory IL-10 was dramatically increased, whereas pro-inflammatory TNF-α and IL-6 were moderately induced in RAW264.7 macrophages stimulated with Tv-ELVs. Mice pretreated with the vesicles had an elevated production of IL-10 but reduced levels of IL-6, IL-13, and IL-17 [1]. The reduction in the inflammatory response by the Tv-ELVs likely favors the persistence of *T. vaginalis* parasites.

Thus, the EVs secreted by *T. vaginalis* parasites can promote the pathogenesis of the disease by modulating the host immune response mediated by the secretion of cytokines and by facilitating the parasites’ adherence and persistence in the host.

### 2.2. Entamoeba histolytica

*Entamoeba histolytica* is an anaerobic parasite that infects the colon. It has a biphasic lifecycle consisting of the dormant cyst stage, which survives in the environment, and a motile trophozoite stage, which causes invasive disease [26]. While most infections are localized to the colon (colitis), extra-intestinal amoebiasis can sometimes occur through invasion of the colonic mucosa by trophozoites, which can spread to the liver and cause amebic liver abscesses. *Entamoeba* is one of the primary causes of parasitic mortality in humans and represents a significant health concern in the developing world. 

*E. histolytica* EVs consist of the characteristic cup-shaped vesicles of around 125 nm in diameter [27]. A second study reported more variation in the size of the amoebic EVs: the amoebic EVs had an average size of 167 nm, but EVs (microvesicles) of up to 600 nm were also observed [28]. Both studies used the same EV isolation protocol (a commercial PEG-based kit) and the variation in size could be due to different culture conditions or variance in the amoeba cultures maintained in the different labs. 

Characterization of the EVs’ proteomes showed similar results in both studies, with selective enrichment of proteins associated with vesicle formation, cytoskeletal proteins, and common EV marker proteins such as elongation factor 1-alpha, heat shock protein 70, and ADP-ribosylation factor [27,28]. However, tetraspanins, which are common EV marker proteins, were not detected in the *E. histolytica* EVs’ proteome in either of these reports. The *E. histolytica* genome has 17 tetraspanins, some of which are highly expressed in the parasites, but none of them was found on the EVs, suggesting a distinct mechanism of biogenesis of these EVs that is not dependent on tetraspanins. 

RNAseq of the EV RNA showed that a 27 nt antisense RNA population was enriched in the EVs [27]. *E. histolytica* parasites contain a 27 nt antisense small RNA (AS sRNA) population that can regulate the expression levels of specific target genes to very low levels [29,30]. Interestingly, the EVs were also found to contain some proteins that have been identified as members of the RNA-induced silencing complex (RISC) in *Entamoeba*, such as Argonaute 2-2 and a Tudor-domain-containing protein. This could suggest that *E. histolytica* EVs might serve to transport these antisense small RNA effector molecules to other cells. 

Recently, the presence of tRNA halves in the *E. histolytica* EVs was also reported. tRNA halves were accumulated in *E. histolytica* parasites during stress [16]. This accumulation of tRNA halves was seen to be concomitant with a decrease in protein translation during stress. The packaging of the tRNA halves in the EVs suggests a role for the tRNA halves in modulating gene expression in distal cells. However, more work is required to confirm this hypothesis. As mentioned before, the packaging of tRNA halves in EVs from unstressed cells needs to be confirmed. The usual EV isolation protocols require serum starvation, which would lead to the accumulation of tRNA halves in the parasites and might cause their subsequent packaging in the EVs. 

Amoebic EVs were seen to be instrumental in inter-parasite communication. EVs isolated from encysting parasites promoted encystation in other parasites, whereas EVs from metabolically active trophozoites impeded encystation (these experiments were conducted on *Entamoeba invadens* as an encystation model) [27,31]. Further investigations to identify the cargo content of EVs from encysting cells could help to elucidate the regulatory factor(s) and their roles in parasite–parasite communication.

Díaz-Godínez et al. characterized the immunomodulatory role of the EVs of *E. histolytica* on neutrophils [28]. Amoebic EVs were found to be incorporated into neutrophils and transfer their cargo into the neutrophils following membrane fusion. This resulted in a drastic reduction in the oxidative burst and NETosis from the neutrophils stimulated by PMA, ionophore A23187, or the amoeba itself. Although the amoebic EVs contained ROS and were able to transfer them to the neutrophils, they still had a suppressive effect on the NETosis induced by other stimuli (chemical or biological). The mechanism of the inhibition of NETosis by amoebic EVs are still not understood and further work is required. 

Thus, the data suggest that *Entamoeba* EVs are capable of impacting the parasite’s biology (conversion to cysts) and modulating the host (suppressing the immune response and promoting infection). However, further work needs to be carried out to elucidate the role of these EVs in these biological processes, and the specific biomolecules they carry. 

### 2.3. Giardia

The protozoa *Giardia duodenalis* is the causative agent for the zoonotic enteric infection called giardiasis. The parasite is transmitted by the fecal–oral route through ingestion of the cysts from contaminated water, leading to colonization of the intestine by the trophozoites. The trophozoites attach tightly to the outside of the epithelial cells of the small intestine though their adhesive disks [32]. *Giardia* has undergone reductive evolution and lacks an endosomal/lysosomal system, a Golgi complex, peroxisomes, and mitochondria [33]. Instead, a network of peripheral vesicles (PVs) performs several roles, such as those of multivesicular bodies, including the release of extracellular vesicles. 

Evans-Osses et al. reported that EVs were secreted by *Giardia* in response to different environmental conditions such as pH levels and the concentration of calcium [34]. The vesicles induced in the presence of calcium, a known inducer of MVs, were defined as microvesicles (MVs), since their size was around 200 nm. Consistent with what was seen in other systems, the origin of the *Giardia* MVs was found to be the plasma membrane and required a lipid raft for synthesis [35]. In contrast, Zhao et al. reported that *G. duodenalis* trophozoites secrete cup-shaped vesicles with a membrane bilayer and a diameter of around 100–200 nm and called them EVs [36]. A different group reported two distinct types of EVs secreted by Giardia: small vesicles (SVs) that were smaller than 100 nm, and large vesicles (LVs) that were 100–400 nm in size [37]. The SVs were exosome-like particles, and their biogenesis and protein content were dependent on lipid rafts. Inhibitors of lipid rafts affected the morphology and composition of SVs (and, to a smaller extent, LVs) and disrupted the attachment of the trophozoites to the host cells [37]. 

Analysis of the MVs’ cargo showed the presence of proteins seen in MVs from other systems [34]. Proteins involved in pathogenesis such as VSPs and giardins were found in the MVs, along with a high number of uncharacterized proteins that were found exclusively in the MVs of the cyst stage. The MVs’ cargo also contained small RNA, though this was not sequenced. The MVs were reported to aid in the trophozoites’ attachment to Caco-2 cells, although MVs alone did not affect cell viability (though *Giardia* can induce apoptosis of the host cell) [34]. 

The MVs have a moderate immunomodulatory effect on their internalization by human immature dendritic cells (iDCs). Subsequent studies showed a stronger response of the host cells’ innate immune system after exposure to *Giardia* EVs. Endocytosis of *G. duodenalis* EVs (GEVs) by murine peritoneal macrophages induced inflammatory cytokines such as IL-6, IL-10, IL-12, IL-17, IL-18, IFN-γ, TNF-α, IL-1β, Cxcl2, and Ccl20, which may contribute to the host’s resistance to the development of the disease. Several Toll-like receptors (TLRs) and Nod-like receptors (NLRs) were seen to be upregulated, and TLR2 and NLRP3 inflammasome signaling pathways were activated after treatment with GEVs. Pretreatment of murine macrophages with the GEVs enhanced the inflammatory response to *G. intestinalis* trophozoites [2].

A more recent study characterized the RNA content of the vesicles secreted by *Giardia duodenalis* [38]. The vesicles were found to be similar in size, shape, and protein and lipid composition to those described for exosomes from other eukaryotic cells and were therefore called “exosomal-like vesicles (ElVs)”. RNAseq of the ElVs secreted from different assemblages of *Giardia* showed distinct small RNA populations, including ribosomal small RNAs (rsRNAs), messenger small RNAs (msRNAs), and transfer small RNAs (tsRNAs). No miRNA could be identified in the ElVs. This was expected, since *Giardia* lacks the usual miRNA machinery, such as DROSHA and XPO5 [38]. 

The presence of tRNA fragments in EVs from *Giardia* adds to the growing body of evidence that tRNA fragments are ubiquitously packaged in EVs and suggests a possible role in the modulation of gene expression in the target cells. However, further work would be required to confirm the biological role of tRNA fragments in these EVs. 

Thus, the EVs secreted by *Giardia* can promote the pathogenesis of the disease by aiding the trophozoites’ attachment and internalization. However, exposure of the immune cells to the EVs elicited a pro-inflammatory cytokine response that contributed to resistance against the parasites.

### 2.4. Leishmania

*Leishmania* spp. is the causative agent for a variety of diseases called Leishmaniasis, which is spread by the bite of infected phlebotomine sandflies. Leishmaniasis vary from cutaneous infections to the potentially lethal visceral infections that have a high mortality rate and economic burden globally [39]. *Leishmania* parasites exist as two structural variants in two distinct hosts, depending on the stage of their lifecycle: the *Leishmania* amastigote is the intracellular stage that lives inside macrophages in mammals, while the promastigote is the motile extracellular form in the digestive tract of the vector, sandflies. 

Several reports have characterized the EVs secreted by different *Leishmania* parasites. Both exosome-like [40,41] and microvesicle-like vesicles were observed [42,43]. The EV proteome was analyzed and found to contain immunogenic proteins such as active proteinases, and virulence factors such as HSP100, LPG2, and glycoprotein 63 kDa (gp63) [40,44,45,46,47]. Glyceraldehyde-3-phosphate dehydrogenase (GAPDH) was found to be highly enriched in EVs of *L. major* and contributed to the inhibition of TNF-α [43]. The protein kinase casein kinase 1.2 (CK1.2) was released by the promastigotes’ exosomes in several *Leishmania* species and directly phosphorylated host’s extracellular substrates [48,49,50].

The RNA content of *Leishmania* EVs showed selectively and specifically enriched small RNAs derived exclusively from noncoding RNAs such as rRNAs and tRNAs. Interestingly, tRNA fragments, predominantly tRNA halves (tsRNAs) were found to be amongst the highly enriched RNA populations. The majority of tsRNAs in the EVs of both *L. donovani* and *L. braziliensis* were 5′ tRNA halves. Northern blot experiments demonstrated that the tRNA-Asp and tRNA-Leu were abundant in the EVs, though no detectable amounts were observed in the parental parasite’s RNA [51]. Interestingly, EVs from another parasite, *E. histolytica,* were also reported to contain tRNA-Asp [16]. A recent study has reported that *Leishmania*-derived small RNAs, including tsRNA, can be found in the blood of mice infected with *L*. *donovani* and *L. amazonensis* [52]. The enrichment and dissemination of small RNA through EVs in the host merits the investigation of the regulation of the host’s genes by these parasites. 

EVs play an important role in the *Leishmania* infection cycle in both the promastigote and amastigote stages [48,53,54]. Promastigotes can secrete EVs into the lumen of the sandfly midgut, from where they are egested along with the parasites when the insect bites the mammalian host. Co-inoculation of the EVs with the parasites has been shown to facilitate infection [55]. Moreover, macrophages infected with amastigotes secrete EVs that can modulate innate and adaptive immune responses in the mammalian host [41,54]. 

Martin Olivier’s group published seminal work on the secretion of EVs from *Leishmania* promastigotes in the sandfly midgut, elucidating their role as vehicles for biologically active molecules. Midguts from sandflies infected with *L. infantum* and *L. major* were analyzed by TEM, and vesicles were imaged on the promastigotes’ membrane surfaces [55]. EVs were egested along with the parasites when feeding on blood and were found to exacerbate infection through the induction of inflammatory cytokines such as IL-17a and IL-10. The EV proteome was found to be highly conserved among strains, except for a few specific proteins that were enriched differently in the parental parasites in response to drugs. The EVs contained several virulence factors, transcription factors, and proteins encoded by drug resistance genes [56]. The EVs secreted by drug resistant *Leishmania* parasites were highly enriched in genomic regions containing drug resistance genes, and these EVs were capable of transmitting the drug resistance genes to distal parasites and promoted their survival [57]. In another interesting discovery, they showed that *Leishmania* RNA virus 1 (LRV1) can hijack *Leishmania’s* exosome as a vehicle to reach the extracellular environment. *Leishmania* EVs enabled viral transmission and infectivity by acting as an envelope for LRV1, which protected the virus from RNAses such as RNAseIII [12]. 

The effect of *Leishmania* EVs on the host’s immune response has been studied in detail and was found to vary with species [25]. *L. donovani* EVs were reported to have an immunosuppressive effect, inducing a T helper 1 response in CD4^+^ T cells, promoting the production of IL-10, and inhibiting the production of TNF-α in monocytes [49,58]. EVs from *L. amazonensis* promoted the progression of the disease by increasing the expression of IL-6 and IL-10 by naïve macrophages [54]. Recent work has shown that the EVs secreted by *Leishmania*-infected macrophages (LiEVs) contain parasite-derived molecules and can carry these to the liver in murine models of infection [59,60]. Although there was some initial evidence that LiEVs are associated with the M2 polarization of macrophages, further work is required to verify that the EVs are responsible for the alternatively activation of M2 polarization. 

Weber et al. analyzed the EVs isolated from axenic cultures of the promastigotes of the cutaneous parasites *L. shawi* and *L. guyanensis* [41]. Zeta potential measurements indicated that the EVs were likely to flocculate and integrate with the cellular membranes in a nondisruptive way. Incorporation of the EVs into macrophages facilitated the recognition of parasitic antigens by surface and intracellular pattern recognition receptors (PRR) such TLR4, NOD1, and TLR9. A mix of both pro- and anti-inflammatory cytokines was observed, suggesting that the EVs can promote a balanced and sustained low level of infection. 

Thus, *Leishmania* EVs promote the parasites’ survival and infectivity during Leishmaniasis. However, the impact of these EVs on disease outcomes varies among *Leishmania* species, and both immunosuppressive and immunostimulatory effects have been observed.

### 2.5. Plasmodium

The apicomplexan protozoan *Plasmodium* spp. is the causative agent of malaria, one of the most important infectious diseases in the world. Infection with *P. falciparum* is potentially life-threatening, while *P. vivax* is commonly responsible for less severe forms of malaria. The parasite’s sporozoites are transmitted to humans by the bite of an infected female *Anopheles* mosquito when it feeds on blood. The sporozoites migrate to the liver, infect the hepatocytes, and multiply to release merozoites. During the blood stage, merozoites replicate inside the erythrocytes. Mature gametocytes are released into the bloodstream and can be taken up by the mosquito. Sexual development of the parasites occurs in the mosquito midgut, resulting in ookinetes which penetrate the epithelium and form oocysts. These replicate to form the sporozoites that migrate to the salivary glands to complete the lifecycle [61]. 

During infection, EVs can be released into the body fluids directly by the extracellular sporozoites, and by the erythrocytes and hepatocytes infected by the parasite. At the same time, parasitic infection can modulate the secretion of EVs from activated effector cells [62]. However, research on the EVs related to malaria has predominantly focused on the intraerythrocytic stages of the parasite. High levels of plasma EVs have been reported in patients and are known to contribute to malaria-associated clinical symptoms, such as cerebral malaria [63]. 

EVs from *Plasmodium*-infected RBCs (iRBCs) have been very well characterized and were found to contain a variety of molecules mediating pathogenesis and intercellular communication between the host cells, and between the host and the parasite [64]. Interestingly, *Pf*-iRBC-EVs from RBCs infected with different stages of malaria parasites had distinct protein expression profiles, which indicates the tight control of the EV cargo by the intracellular parasites [65]. Different proteomic analyses have confirmed that virulence-associated proteins such as PfPTP2 and MSP-1 are enriched in these EVs, and they were shown to have a role in EV-mediated communication [66,67,68]. In a recent report, Abou Karam et al. identified two EV subpopulations derived from *Pf*-iRBC with distinct size ranges of 30–70 nm and 70–300 nm. The proteomes of these two populations were distinct, which indicated that these EVs could induce different phenotypic effects in separate recipient cells [69]. 

The functional role of the nucleic acid content of *Pf*-iRBC EVs has also been characterized well. It was reported that the EVs mediate cell–cell communication between the infected erythrocytes by delivering genes, and this was found to promote their differentiation to gametocytes [67]. The DNA content of the EVs also has a role in altering the host’s gene expression: *Pf*-iRBC EVs carry *P. falciparum* gDNA, which, when internalized by monocytes, is recognized by the host’s PRRs, which triggers a signaling cascade, resulting in modulation of the induction of the host’s genes [70,71]. 

*Pf*-iRBC EVs can also carry small RNA molecules to distal cells. In an elegant study, Mantel et al. showed that the EVs contain functional miRNA derived from the host’s RBCs. The miRNA–Argonaute 2 complexes in the EVs could modulate the expression of the target gene and the properties of the barrier in target endothelial cells [72]. Small regulatory RNA populations from these EVs consisted of mRNAs coding for exported proteins and proteins involved in drug resistance, as well as noncoding RNAs, such as rRNAs, small nuclear (snRNAs), and tRNAs. tRNA-derived fragments from both the host and the parasite were identified in the EVs, with tRNA halves being the predominant species [73,74]. 

The role of *Pf*-iRBC EVs in helping the parasite evade the host’s immune mechanism by regulating the cytokine levels from various immune cells has been reviewed extensively before [62,64,75]. The pro-inflammatory response (characterized by the activation of monocytes and macrophages, and the secretion of pro-inflammatory cytokines such as IL-6, IL-12, and IL-1β) has been most commonly reported. Recently, these EVs have also been reported to prime naïve RBCs to enable invasion through a 20S proteasome secretion mechanism [76]. *Pf*-iRBC EVs have also been shown to suppress the immune response under certain situations, indicating the complex and dynamic role of these EVs [77]. 

*Pf*-iRBC EVs have also been proposed to have a role in quorum sensing (QS) by the parasites by regulating their growth density. Plasmodium EVs could induce apoptosis by transporting *Pf*LDH to the recipient cells if the parasite population was high [78]. 

Several studies have been carried out to investigate *Plasmodium* EVs, and there is mounting evidence for their role in driving the progression of the disease. The functions of different biomolecules transported inside the EVs have been elucidated, and they were found to contribute to a complex interplay between the parasite and the host’s immune system. 

### 2.6. Toxoplasma

The obligate intracellular parasite *Toxoplasma gondii* infects nucleated cells in humans and animals, leading to toxoplasmosis, an important zoonotic disease which causes a significant economic burden worldwide.

EVs have been reported from extracellular tachyzoites of *T. gondii.* Both exosome-like and microvesicle-like EVs were identified. A fraction of these vesicles was morphologically similar to mammalian exosomes, with a size of around 138–170 nm. These EVs were found to contain miRNA and were found to activate murine macrophages to induce TNFα, iNOS, and IL-10 [79,80]. In a different study, EVs from tachyzoites were found to induce several changes in the host’s cells that helped enhance the survival, transmission, and progression of *T. gondii* parasites [81]. Interestingly, immunization with EVs from *T. gondii* tachyzoites could confer protection in murine infections, activating cellular and humoral responses, and could elicit a combined Th1 and Th2 profile. The immunization reduced parasitemia and increased the survival index [82]. 

EVs from cells infected with *T. gondii* have also been well characterized [83]. The parasite can package its proteins into the host’s EVs, which helps protect the parasite from the host’s immune system. Proteomic analysis of the EVs from human foreskin fibroblasts (HFF) infected with *T. gondii* identified classical EV marker proteins such as extracellular matrix glycoproteins, calcium-binding proteins, filament-associated proteins, members of the annexin family, HSP 70, and CD63 [84]. RNA characterization of these EVs showed the enrichment of mRNA and miRNA. Four specific mRNA species with neurologic activity, namely Rab-13, eukaryotic translation elongation factor 1 alpha 1, thymosin beta 4, and an LLP homolog, were the most predominant [85]. 

*T. gondii* EVs were seen to impart protective immunity in both *in vitro* and *in vivo* studies. EVs from infected dendritic cells of the host had antigen and adjuvant properties, and could confer protection against *T. gondii* in mouse models [86,87]. Moreover, immunization of BALB/c mice with *T. gondii* exosomes improved the survival time, with both humoral and cellular responses [88]. 

Recently, Tedord et al. described a novel type of the regulation of neurotransmission mediated by EVs from *T. gondii* that may contribute to maintaining chronic infection. *T. gondii* EVs from infected noradrenergic cells were shown to contain a natural mammalian antisense lncRNA that induced gene silencing and DNA hypermethylation *in vitro* and in the brain [89]. 

### 2.7. Trypanosoma cruzi

The obligate intracellular parasite *Trypanosoma cruzi* is the causative agent for Chagas disease (CD) or American trypanosomiasis. 

EVs from *T. cruzi* epimastigotes were first described by da Silveria et al. in 1979 [90]. Later, these EVs were characterized and found to consist of two populations with distinct sizes. Proteomic analysis showed proteins involved in metabolism, host–parasite interactions, signaling, nucleic acid binding, parasite survival, and virulence factors such as trans-sialidases, mucin, mucin-associated surface protein, cruzipain, and phosphatases [91,92,93]. Distinct proteome profiles were observed in EVs from different strains of the parasites and corresponded to different infection profiles in these strains [94]. 

Characterization of the RNA content of the EVs showed the enrichment of tRNA halves as well as the argonaute TcPIWI-tryp protein, suggesting an endocytic/exocytic pathway in *T*. *cruzi* [95,96]. The EVs deliver tRNA halves to the recipient parasites as well as the mammalian host’s cells. The EVs secreted by epimastigotes were seen to strongly promote metacyclogenesis (transformation of the epimastigote to the trypomastigote form) in axenic cultures [97]. Garcia et al. also found that *T. cruzi* EVs elicit changes in the gene expression of recipient HeLa cells through a microarray analysis. Genes related to the host’s cell cytoskeleton, extracellular matrix, and immune responses pathways were found to be differentially expressed upon EV treatment. Interestingly, the parasitic tRNA halves packaged in the EVs were identified as effector molecules regulating gene expression in the HeLa cells. Overexpression of tstRNA_Thr in HeLa cells resulted in upregulation of the target CXCL2 gene [96]. Recently, Cornet-Gomez et al. carried out a transcriptomic analysis of Vero cells stimulated by EVs derived from the trypomastigote stage of *T. cruzi* and found the overexpression of genes related to ubiquitin-related processes and downregulation of Rho-GTPase. The authors also reported the anti-apoptotic role of these EVs [98]. 

EVs released by several *T. cruzi* strains were shown to trigger an proinflammatory response mediated by TNF-*α*, IL-12, IL-6, and NO which enhance the invasion of the host cells via Toll-like receptor 2 [99], although the secretion of anti-inflammatory IL-10 from T and B cells has also been reported [25].

### 2.8. Trypanosoma brucei

*Trypanosoma brucei* is the causative agent of human African trypanosomiasis (HAT), also known as sleeping sickness.

*T. brucei* EVs were first described by Szempruch et al. The EVs were found to be 70–80 nm in size and were formed by budding of the flagellar membrane. Proteomic analysis of the EVs identified several flagellar proteins that contribute to virulence. Trypanosome EVs were found to fuse with mammalian erythrocytes, resulting in rapid erythrocyte clearance and anemia [100].

Eliaz et al. showed that *T. brucei* EVs had a role in parasite–parasite communication and could affect a change in the migration of the recipient parasites. EVs from stressed trypanosomes were incorporated in the recipient parasites and affected their social motility by causing repulsion. Thus, the parasites were proposed to signal to other parasites about immediate stressful environments [101]. 

### 2.9. Naegleria

*Naegleria fowleri*, known popularly as the “brain-eating amoeba”, is a thermophilic free-living amoeba that is the causative agent for the fulminant disease known as primary amoebic meningoencephalitis (PAM). *N. fowleri* does not require a host in nature or a vector for transmission; however, it can thrive in the environment, particularly in warm rivers and lakes [102].

The EVs secreted by *N. fowleri* have a mean size of around 206 nm. Preliminary proteomic analysis revealed a predominance of serine proteases in the EVs. [103]. A different study characterized *N. fowleri*-derived microparticles (*Nf*-MP) and *N. fowleri*-derived exosomes (*Nf*-Exo), although the sizes of these two were not too different, with an average diameter of 150 nm and 140 nm, respectively [104]. The EVs elicited an immune response that was distinct from that known to be induced by the *N. fowleri* trophozoites. Macrophages and microglia cells play an instrumental role in the immune response to *N. fowleri,* though the infection leads to necrosis and apoptosis in these cells [105]. However, no apoptosis was observed in THP-1 monocytes and macrophages exposed to the Nf EVs. 

Stimulation with Nf EVs was seen to activate Thp-1 macrophages and induce the production of IL-8. This was proposed to trigger the migration of neutrophils, leading to their accumulation in the brain and their involvement in the pathogenesis of PAM [104]. 

Further work is required to elucidate the role of *Naegleria* EVs in the progression and pathogenesis of the disease. 

Extracellular vesicles play several important biological functions during the pathogenesis of protozoan diseases. With their cargo of biologically active molecules protected by a lipid bilayer, EVs are the ideal vehicle to communicate with distal cells. Various studies have identified different effector molecules that mediate the signals, including proteins, nucleic acids, and even reactive oxygen species (ROS). Proteins, such as the GP63 protease found in *Leishmania* EVs and the PfPTP2 packaged in *Plasmodium* EVs, play a pivotal role in transmitting signals to the recipient cells [45,47,67]. Small RNA species, including miRNA, antisense RNA, and tRNA halves (tsRNAs), can regulate gene expression in the target cells, as well as initiate the host’s immune response. EVs have also been shown to communicate with the target cells through their DNA cargo. *Plasmodium* EVs can carry drug resistance genes to the recipient parasites and can also regulate gene expression in the host’s cells [73]. Through these manipulations, EVs can also facilitate adherence to the host and promote the progression of the disease. 

It is not surprising, therefore, that EVs are emerging as important mediators of the immune response for various parasites. EVs carry immunogenic molecules that can be identified by pattern recognition receptors (PRRs) and activate the host’s innate immune responses. In contrast, EVs can also carry molecules that have an immunosuppressive effect (e.g., the agonists of PRRs) to have an overall immune-protective effect. Moreover, the specific packaging of these molecules allows for a finely nuanced modulation of the host’s immune response, leading to the secretion of anti-inflammatory or pro-inflammatory cytokines by the same parasite, depending on its situation. 

On the basis of these properties, EVs’ functions can be broadly classified into three main categories.

1. Parasite–parasite communication. EVs can coordinate the interactions between parasitic cells. *E. histolytica* EVs from encysting parasites were shown to carry encystation signals to trophozoites and promote stage conversion [27]. Similarly, *T. vaginalis* parasites can transfer adherence-promoting factors to other parasites through EVs [21]. RBCs infected with *P. falciparum* secrete EVs that can carry signals for the differentiation of gametocytes or drug resistance to other parasites [67]. *T. brucei* parasites can transmit factors that promote invasion and migration [101].
2. Parasite–host interactions: EVs can also benefit parasites by regulating the host’s cells to promote infection. *P. falciparum*-infected RBCs secrete EVs which can carry small RNA such as miRNA and tsRNA to the endothelial cells [72]. Similarly, tsRNA in EVs from *T. cruzi* parasites have been shown to regulate mRNA levels in HeLa cells [96]. *T. gondii* EVs carry antisense RNA to downregulate gene expression in murine brain models [89]. *Giardia* EVs promote attachment to the host’s cells [34].
3. The role of EVs in immunomodulation has been extensively studied, and both pro-inflammatory and anti-inflammatory functions have been observed. For example, *T. vaginalis* EVs stimulate the host’s macrophages to secrete pro-inflammatory cytokines such as IL-6 and IL-8, which help promote the infection [21]. *P. falciparum*-infected RBCs secreted EVs with a complex role in promoting the pathogenesis of the disease by eliciting the production of cytokines and immunosuppression in various immune cells of the host [62,64,75]. *Trypanosoma* and *Leishmania* EVs also facilitate the progression of the disease by immunosuppression [53,58,99]. *T. cruzi* EVs have been observed to stimulate the secretion of both pro- and anti-inflammatory cytokines, and consequently play various regulatory roles in the host’s cells [106]. However, sometimes, EVs can also stimulate the host’s immune response and promote clearance of the disease by the host. *Giardia* EVs stimulate the secretion of IL-1β, IL-6, and TNF-α, which enhance protection against the disease [2]. EVs from *N. fowleri* stimulate the production of IL-8 from the macrophages that would affect the pathogenesis of the disease [104].

## 3. Conclusions

The data demonstrate that EVs play a central role in the interplay between parasites and the host cells. Parasites control and modulate the cargo of not only the EVs secreted directly by them into the extracellular environment, but also of the EVs secreted by the host cells they infect. This ability to manipulate the content of host-derived EVs grants a distinct advantage to the parasites concealed within the host cells, enabling them to wield their influence over distant target cells. 

Recent research has identified newer effector molecules in the cargo. Functional small RNA species such as tsRNA and miRNA, along with associated proteins such as argonautes, could help regulate the gene expression profiles in distant cells. 

EVs initiate a spectrum of immune responses from the host and are capable of inducing both pro-inflammatory and anti-inflammatory effects. Several studies have provided evidence that the cargo inside EVs is dynamically regulated by the parasites in adapting to their immediate environment and conditions. Therefore, the same parasitic EVs can elicit different immune responses at different stages of the lifecycle, indicating the complex and nuanced modulation of the host’s immune response. 

Understanding the precise role of EVs in the physiological and natural contexts demands more sophisticated approaches. Despite these challenges, the study of EVs in parasite–host interactions holds great promise in advancing our understanding of infectious diseases and potential therapeutic targets.

## Data Availability

Data sharing not applicable.
